# Increasing weight-bearing physical activity and calcium-rich foods to promote bone mass gains among 9–11 year old girls: outcomes of the Cal-Girls study

**DOI:** 10.1186/1479-5868-2-8

**Published:** 2005-07-19

**Authors:** Simone A French, Mary Story, Jayne A Fulkerson, John H Himes, Peter Hannan, Dianne Neumark-Sztainer, Kristine Ensrud

**Affiliations:** 1 Division of Epidemiology and Community Health, University of Minnesota, Minneapolis, Minnesota, USA; 2 School of Nursing, University of Minnesota, Minneapolis, Minnesota, USA

## Abstract

**Background:**

A two-year, community-based, group-randomized trial to promote bone mass gains among 9–11 year-old girls through increased intake of calcium-rich foods and weight-bearing physical activity was evaluated.

**Methods:**

Following baseline data collection, 30 5th-grade Girl Scout troops were randomized to a two-year behavioral intervention program or to a no-treatment control group. Evaluations were conducted at baseline, one year, and two years. Measures included bone mineral content, density, and area (measured by DXA), dietary calcium intake (24-hour recall), and weight-bearing physical activity (physical activity checklist interview). Mixed-model regression was used to evaluate treatment-related changes in bone mineral content (g) for the total body, lumbar spine (L1-L4), proximal femur, one-third distal radius, and femoral neck. Changes in eating and physical activity behavioral outcomes were examined.

**Results:**

Although the intervention was implemented with high fidelity, no significant intervention effects were observed for total bone mineral content or any specific bone sites. Significant intervention effects were observed for increases in dietary calcium. No significant intervention effects were observed for increases in weight-bearing physical activity.

**Conclusion:**

Future research needs to identify the optimal dosage of weight-bearing physical activity and calcium-rich dietary behavior change required to maximize bone mass gains in pre-adolescent and adolescent girls.

## Background

Osteoporosis currently affects over 25 million people in the United States [1,2] and low bone mineral density (BMD) is a major factor involved in bone fractures in postmenopausal women and the elderly. [3,4] Variation in bone mass accumulation during childhood and adolescence may be an important determinant of the risk of sustaining osteoporotic fractures during later adulthood, since 45% of the adult bone mass is built and enlarged during adolescence. [4,5] Thus, maximizing bone mass in youth may be one of the most effective preventive strategies available. [5]

Although an estimated 60–80% of the variance in peak bone mass (PBM) is attributable to genetic variables, [6-9] eating and physical activity behaviors represent modifiable variables that make small but significant contributions to the attainment of (PBM). [10] From a population perspective, small increases in PBM achieved through eating and physical activity behavior changes could significantly decrease the population-based fracture risk later in life. [10]

To date, the majority of primary prevention interventions for increasing bone mineral mass among children and adolescents have focused on increasing calcium intake through calcium supplementation from pills or fortified foods. [11-15] The study design of these primary prevention trials has been individual-randomized trials with no behavioral change intervention component. In general, results of these studies have shown positive associations between calcium (CA) supplementation and increases in bone mass. [11-16] However, bone mass gains are not sustained once supplementation ceases. [11,12,16]

Several physical activity primary prevention trials have been implemented in school-based settings (for example in physical education classes) and have randomized either individuals or classrooms. [17,18] Similar to the CA supplement trials, however, the studies have not included a behavioral change intervention component. Results of the available studies suggest a positive impact of weight-bearing physical activity (WBPA) on bone mass gains in children, particularly in the femoral neck, spine and total body. [16,19,20] However, little is known about the amount and type of physical activity (PA) needed to increase BMD gains or whether there is a threshold or dose-response relationship.

It has been hypothesized that WBPA and CA intake may produce additive or interactive effects on bone mass gains. We are aware of only one randomized controlled trial that simultaneously targeted increased intake of CA-rich foods and WBPA to evaluate bone mass gains among children. [20] Sixty-six eight-year old girls were randomized to either a low-impact or high-impact PA class during school (three times per week for 20 minutes), and consumed either CA-fortified foods or the same unfortified foods for 8.5 months. No behavioral change intervention component was included. Results showed that the effects of increases in CA intake and WBPA on rate of bone gain varied at different skeletal sites. Clearly, additional research is needed to clarify the independent and interactive effects of each bone-related behavior, WBPA and CA intake, on site-specific and generalized bone mass gains in girls. Furthermore, community-based studies will provide information about the effectiveness of behaviorally-based, primary prevention interventions that are implemented in settings that are widely generalizable.

The present paper describes the primary outcomes of a community-based, group-randomized trial to increase bone mass gains among 9–11 year-old girls by targeting increases in intake of CA-rich foods and WBPA. It was hypothesized that the behavioral intervention would result in increases in dietary CA intake and WBPA, and in the rate of bone mass accrual. Innovative aspects of the present study included 1) its focus on changing eating and PA behaviors to increase bone mass growth; 2) its focus on pre-adolescent girls, a group at risk for declines in CA intake and PA levels as they develop into adolescence; and 3) its unique collaboration with a community-based organization (the Girl Scouts of America) as a channel to implement a health behavior intervention.

## Methods

### Study Overview

Thirty 5th-grade Girl Scout troops were recruited and randomized to a two-year behavioral intervention program (n = 15 troops) or to a no-treatment control group (n = 15 troops). The behavioral program was implemented during 5th and 6th grades by trained troop leaders as part of the regular troop meetings. The intervention program was based on Social Cognitive Theory [21] and consisted of ten 90-minute activity-based sessions during each of the two years. It focused on the development of behavioral skills to choose CA-rich foods and to engage in WBPA (see Table 1). Behavioral goals for the intervention were to increase daily dietary CA intake to 1300 mg/day (increase of 4 daily servings of CA rich foods; about 800 CA mg/day) and to increase WBPA to 120 minutes per week. A continuously available, interactive web-based program, and a one-week summer camp between 5th and 6th grade years, were implemented as components of the intervention program. Parents also were targeted through the web-based program. Control troops did not receive any program and conducted their usual troop meeting activities during the two-year intervention period. Evaluation was conducted with individual girls and a parent at clinic visits at baseline prior to randomization, at one-year follow-up, and at the end of the study (two year follow-up).

**Table 1:** CAL-GIRLS INTERVENTION PROGRAM *(Selective Version)*

**INTERVENTION COMPONENT**	**5^th ^GRADE**	**6^th ^GRADE**
**TROOP MEETINGS ***(behavioral curriculum)*	Calcium Matching Game Bone Bead Bracelets CAL-Girls Club Radio Show Bone Builder Breaks CAL-Girls Web Site Grocery Store Scavenger Hunt Calcium-rich Food Sculptures Jump Rope w/U of M Staff Bone-opoly Game Creating Calci"yum" Cereal Treats CAL-Girls Time Capsules "Got Milk?" Mustaches & Photos CAL-Girls Parent Party	Digiwalkers CAL-Girls Web Site CAL-Girls Smoothies CAL-Girls Makin' Movies & Filming at MTN Studio Decorate CAL-Girls T-shirts Fruit & yogurt parfaits/sundaes CAL-Girls on the Grow! Game CAL-Girls Are Great! Booklets Aerobic Step-building Workshop YWCA/YMCA Step-Aerobics Class Calcium-rich Party dips & snacks Step Right Up! Parent Party

**CAL-Girls HOME ACTIVITIES**	***Under Our Roof Activities *** • Find 5 calcium-rich foods in your house. Place a CAL-Girls sticker on each item. • Explore the CAL-Girls Web Site and post a message to Calcy the Cow. • Spend at least 20 minutes each week for two weeks doing Bone Builder Activities with your family. • Create three calcium-rich meals with your family.	***CAL-Girls Challenge Cards ***Examples: • A*dd your favorite kind of cheese to a snack, soup or main dish.* • *Ask an adult what calcium-rich foods they ate today* • *Visit a fitness trail. Spend 20 minutes doing Bone Builder physical activities.* • *Ask adults in your family to come up with FIVE reasons why it's important to eat Bone Builder Foods.*

**CAL-Girls WEB SITE**	Calcium News Bone Building Activities Web Site Fun Virtual Grocery Store All About Cheese! The Cow Connection In Your Community Sing, Shake, Celebrate Mostly Milk Is It Over?	Welcome Back! Big News on Bones Spread the Word Time for Bones! The Miracle of MILK! Bigger, Better & Stronger Bones! Step Up for Strong Bones! "Y" Strong Bones What's Your Calcium Sign? Bones Last a Lifetime!

CAL-Girls SUMMER CAMP*	**COHORT ONE (2001)**	**COHORT TWO (2002)**
	Jump Rope Recycling & The Environment Ice Cream in a Bag Farmer's Market Creating Cereal Necklaces Making Pizza Homemade Fruit Leather or Yogurt Pancakes Swimming Camp Olympics	Jump Rope Germs & Hand-washing Food Group Bingo Food Jeopardy Milk, and Sugar in Soda Pop Milk Crossword Puzzle Ice cream in a Bag Making Sun Pizzas Swimming Autograph Books

*NOTE: The CAL-Girls Summer Camp was offered in the summer between 5^th ^and 6^th ^grade.

### Troop Recruitment

The study was conducted in collaboration with two local councils of the Girl Scouts of America. Girl Scout Council staff were active collaborators in the program development and implementation. Troops from the Minneapolis and St Paul metropolitan area were recruited to take part in the study through mailed fliers to troop leaders and troop leader meeting announcements. Troop eligibility criteria were: 1) troop size = 8 girls; 2) parental consent and girl assent from each troop member to participate in troop meetings with intervention program activities; and 3) troop plans to remain together at minimum two more years. Troops that met eligibility criteria were scheduled for a recruitment presentation by the study Project Director or the Principal Investigator at a regularly scheduled troop meeting. Recruitment visits included a brief video showing several Girl Scouts from our pilot study completing clinic measures and the pilot Girl Scout troops engaging in intervention activities. A written study description and consent forms were distributed and discussed at the recruitment meeting. Parents were invited to ask questions and were asked to sign the consent form at the meeting. Girls were asked to sign assent forms. The study was reviewed and approved by the University of Minnesota Research Participants Internal Review Board.

### Intervention Program Implementation

The intervention program was implemented by trained troop leaders with support from research staff. Troop leaders assigned to the intervention group were trained by research staff two times per year during each of the two intervention years. Most training sessions were held at the Division of Epidemiology and Community Health. The first training session focused on intervention activities for sessions one through five, and the second training session focused on intervention activities for sessions six through ten. Research staff reviewed the intervention session content, demonstrated activities with troop leaders, and answered questions. All materials and supplies needed for implementing the intervention activities were provided to troop leaders. Grocery store gift certificates were provided to troop leaders to cover the costs of the CA-rich snacks that were purchased for each intervention session.

Research staff assisted with the intervention program implementation in several ways. Research staff conducted all sessions in which the girls received training on accessing and completing the web-based activities, and were responsible for creating and managing the website, and responding to girls' and parents' queries regarding web program access. Research staff also were responsible for implementing the home-based intervention activities, community-based intervention activities and the summer camp.

### Clinic Visit

All evaluation data were collected by trained research staff during clinic visits held at the University of Minnesota. Clinic visits were conducted at baseline, prior to randomization, and after the first and second years of the study (a total of three clinic visits). Girls were scheduled individually with a parent. Clinic visits required about two hours to complete. Ninety-five percent of the girls who were enrolled in the troops (n = 322 / 340) completed the baseline clinic visit. Reasons for non-completion included repeated scheduling problems (n= 6) and refusal due to concerns about the bone scan (n= 12). All data collectors and bone densitometry technicians received standardized training and met certification criteria prior to collecting participant data. Bone densitometry technicians were state-certified to conduct radiology scans, and also received study-specific bone densitometry training.

### Measures

#### Bone Mass

Bone mass was measured using dual-energy x-ray absorptiometry (Hologic, Inc; QDR-4500; Waltham, MA). Change in bone mineral content (BMC) was the primary outcome variable for the study because it best reflects bone mass change in developing children. [22] Bone mineral density (BMD) and bone area (BA) were also measured and analyzed, and are presented for descriptive comparison with the published literature. Standard positioning protocol and software was used to complete and analyze the scans. [23] BMC (gm) was measured for the total body and at the lumbar spine (L1-L4), total hip, including one sub-region (femoral neck), and one-third (distal) radius. Separate scans were conducted for total body and for each sub-region. At all data collection periods, for hip scans, the femoral neck box was kept constant. However, at follow-up assessments, if necessary, the hip region of interest (ROI) was enlarged to allow for growth. Unossified vertebral transverse processes were outlined manually. A high correlation (r = .98) was observed between the two repeated baseline scans for participants in cohort one (n = 193). Therefore, each site was scanned only once for participants in cohort two (n = 129) and for subsequent cohort scans. Body composition calculations for fat mass (g), lean mass (g), and percentage of body weight that is fat were derived from DXA measures.

Ten inter-operator reliability scans at each bone site were collected during each data collection period. Averaged across assessment periods, BMC reliability was high (whole body r = .995; spine (L1-L4) r = .991; total hip r = .988; femoral neck r = .975; 1/3 distal radius r = .983) and percent bias was approximately 1% at each site. For additional quality control, each technician reviewed the scans measured by the other technician. Scan interference such as gross movement (total body) or casts (radius) eliminated data from at least one of the five scans for 40 girls at baseline and 10 girls at either follow-up period. The lumbar spine system phantom was scanned daily to maintain quality control, and the coefficients of variation (CV) for our machine was as follows: BMC CV = 0.61%; BMD CV = 0.39%; BA CV = 0.47%.

#### Calcium Intake

The Nutritional Data System for Research [24] from the University of Minnesota' s Nutrition Coordinating Center was used to collect information regarding dietary intake. Dietary intake was measured by trained and certified dietary interviewers using a single 24-hour recall with a multiple-pass approach. A single recall was used instead of multiple recalls because the purpose of the recalls was to estimate group means, not individual intake. [25,26] Upon completion of the interview, the girls' parent was asked specific questions regarding the type and dose of vitamin/supplement intake, particularly CA supplements and CA-fortified products. A certified nutrition coordinator reviewed all recall data for quality assurance. Data on dietary energy and dietary CA intake (without supplements) were examined.

#### Weight Bearing Physical Activity

Weight bearing physical activity was measured using the Physical Activity Checklist Interview (PACI). [27] The PACI is an interview-administered instrument to assess the previous day recall of 24 physical activities. Among children, moderate validity has been reported for the PACI when compared to objective measures of PA with a correlation of .51 between the PACI and a heart rate index, and a correlation of .33 between the PACI and an accelerometer. [27] Girls reported minutes spent in each activity during three day-parts (morning, afternoon, evening). Intensity of activity was estimated by girls' self-report of how out of breath they were during each bout of the activity reported (none of the time, some of the time, or most of the time). MET values were assigned to each of the 24 activities [28] and were weighted based on self-reported intensity. [29]

Physical activities were defined by a consensus among the study investigators as either weight-bearing or non-weight-bearing. WBPA behaviors were judged to exert significant gravitational force and inertial impact on the body (e.g., jumping rope, jogging, aerobic dance), and classification was consistent with other research that measured the vertical ground reaction forces of physical activities among youth. [30] The only activities that were not included in the WBPA calculation were: swimming, bicycle riding, and household chores. A measure of osteogenic (bone-forming) PA was calculated to quantify the dose of bone-forming PA reported. [31,32] Osteogenic scores were calculated as: Ground Reaction Force × [log_e_(minutes +1) × (sessions per week -1)]. [32]

#### Weight and height

Weight and height were measured by trained and certified technicians following a standardized protocol. [33] Weight was measured to the nearest .1 kg on a calibrated balance-beam scale. Height was measured to the nearest millimeter using a wall-mounted stadiometer; two height measurements were taken and averaged for data analyses. Girls were measured in cloth gowns without shoes. Body mass index was calculated as weight (kg)/height (m)^2^.

#### Body Composition

Lean body mass (g) and body fat mass (g) were assessed by the DXA scan. Lean mass (without bone) and fat mass values were translated to kilograms (kg) and divided by body weight (kg) to compute percentage body lean and percentage body fat.

#### Sexual maturation

Sexual maturation was measured using direct observation by trained female staff. Breast and pubic hair development were rated separately as stages 1–5 according to standardized procedures. [34] Girls also completed two self-report measures of pubertal development: one using a pictorial format based on Tanner staging [35,36] and one using a series of questions about the girl's physical development from two published studies. [37,38] Approximately one-third (35%–46%) of girls declined to complete the observed pubertal staging protocol at one of the three data collection points. Therefore, a multiple imputation procedure was used to estimate the missing observed values (breast and pubic hair stages separately) from their available self-reported physical development data (i.e., rating of maturity, overall rating of physical development, breast growth, rating of body build), measured height and weight, and age (specific items and procedures can be obtained from the authors). For analyses, imputed data were only used when observed data were not available. Imputation was used in 33% of the 338 girls for both breast development and pubic hair.

#### Medical History Information

At each data collection point, parents reported their child's history of diseases and conditions that might affect bone development. Girls' data were flagged for the following diseases/conditions: bone-related diseases, daily use of steroids known to affect bone metabolism, multiple broken bones, treated growth problem, or other medical conditions (e.g., Turner's syndrome, cerebral palsy).

#### Demographic Information

Demographic Information was collected via questionnaire from the parent who accompanied the girl to the clinic. Ninety-two percent of the accompanying parents were the girls' mothers; 7% were fathers; and 1% were other female guardians. Parental education, annual household income, parent and child race/ethnicity, and marital status were self-reported.

### Process Evaluation Data: Troop Leader Implementation Fidelity and Troop-Level Intervention

#### Participation

Data on troop participation and troop leader implementation were collected from several sources.

#### Fidelity of Implementation

Fidelity of Implementation was measured based on direct observation of troop meetings by trained research staff, who observed 60% of all sessions (six of ten sessions in grade 5 and six of ten sessions in grade 6). At each session, staff observed 1) the troop leader's program material coverage; and 2) whether troop leaders verbally encouraged girls during the troop meetings to do WBPA and eat CA-rich foods outside the troop meetings.

#### Troop Participation

Troop Participation was measured based on four troop-level indicators: 1) research staff observations of the proportion of girls who attended selected troop meetings (six of ten sessions in grade 5; six of ten sessions in grade 6); 2) proportion of home activities completed by the girls in both years of the intervention; 3) web program participation (proportion of web program activities completed by the girls);and 4) proportion of girls in each troop attending the summer camp. These data were summarized by troop to create a troop-level participation score.

### Statistical Analysis

All statistical analyses were conducted using SAS (SAS Release 8.2). [39] The primary outcome analysis was change in BMC and was conducted using mixed-model regression. One common model was developed for all analyses. However, each bone site (total body, spine (L1-L4), total hip, femoral neck, and one-third distal radius) and bone mass outcome [BMC (gm); BMD (gm/cm^2^) and BA (cm^2^)] was analyzed separately. For the mixed-model regression, individual girls were nested within troops and troops were nested within treatment condition. Treatment condition was the primary independent variable. Because the study is a randomized controlled trial, the outcome may legitimately be analyzed without adjustment for covariates. However, covariate adjustment is likely to be necessary in a group-randomized trial because the small number of troops to be randomized increases the probability that the realized randomization will not balance confounders fully. This is especially true when the area is a growth outcome as the girls enter puberty. Moreover, adjusting for measures of maturation increases the precision of the estimate of the dependent variable.

The following covariates were included in the bone mass outcome models: baseline menarcheal status (no/yes), and change in menarcheal status over the two years ("no change" or "achieved menarchy"); baseline height and change in height at interim and follow-up; baseline Tanner stage breast development and change in Tanner stage of breast development (categorical: no change, +1, +2 or +3 stages advanced from baseline), baseline weight, baseline age, and race (reference group: Caucasian). The change in weight was not included as it was thought that the intervention might have an impact on the rate of change in weight. The interim and final measures (adjusted for baseline) were treated as correlated outcomes within girl (ie., repeated measures). The troops were treated as two random effects, one for level and the other for change in troop means as correlated outcomes. Because the intervention was applied to the intact social groups (the troops), the proper error for assessing the effect of intervention is variation at the level of the changes in troop means (i.e., based on the component of variance identified as the troop-by-time interaction). [40]

A similar mixed-model regression was developed to examine secondary behavioral outcome variables: changes in dietary CA intake and WBPA. Models in which change in dietary CA intake was examined were adjusted for total energy intake in addition to the covariates listed above. Height and change in height were not included as covariates in the analysis of change in WBPA or CA intake.

All thirty troops remained in the study for the entire study duration (i.e., troop-level retention was 100%). Ninety-five percent of the girls enrolled in the 30 troops at baseline completed the baseline clinic evaluation (322/340). Of this group, 300 completed the second clinic visit, and 296 completed the third clinic visit. Overall, the individual retention rate was 92%. In addition, 16 girls joined a troop within the two years after baseline; seven girls completed the second and third clinic visits, two girls completed only the second clinic visit, and seven girls completed only the third clinic visit. Data from these girls were included in the calculation of troop means used in the analysis.

Girls were flagged for reporting a lifetime history of bone-related diseases or medical conditions that may affect bone metabolism (e.g., daily steroid use, treated growth problem), or a condition that may inhibit participation in the intervention (e.g., Turner's syndrome, cerebral palsy). Twenty-three, fourteen and twelve girls were flagged accordingly at baseline, one-year and two-year follow-up visits, respectively. Analyses conducted without including data from these girls showed materially identical results and therefore are not presented.

## Results

### Descriptive Characteristics

Table 2 shows the baseline demographic, physical and behavioral variables of the troops by treatment group condition. Parents of participating girls were mostly white, married, college-educated and upper-middle income.

**Table 2:** Baseline and follow-up demographic and physiological variables among 30 Girl Scout troops (troop-level means) by treatment group (n = 15 control, n = 15 intervention)

Label	Baseline		Follow-up 1		Follow-up 2		Two-yr Intervention Effect	
	C	I	C	I	C	I	Effect^A^	SE

Demographics								
Girls' race (% white)	90	90	--	--	--	--	--	--
Family income (% > $90 k)	51	46	--	--	--	--	--	--
Parent's education (% = college degree)	50	59	--	--	--	--	--	--
Girls' age (years)	10.5	10.4	11.5	11.4	12.4	12.3	0.04	0.02
Physical Development								
Breast Stage 1 (%)	37	47	30	40	1	1	--	--
Breast Stage 2 (%)	55	51	53	55	24	23	--	--
Breast Stage 3 (%)	7	2	17	5	44	53	--	--
Breast Stage 4 (%)	0	0	0	0	21	16	--	--
Breast Stage 5 (%)	0	0	0	0	10	7	--	--
Pubic Hair Stage 1 (%)	62	73	23	34	5	7	--	--
Pubic Hair Stage 2 (%)	24	21	33	33	25	23	--	--
Pubic Hair Stage 3 (%)	13	6	28	25	29	38	--	--
Pubic Hair Stage 4 (%)	2	0	15	9	38	31	--	--
Pubic Hair Stage 5 (%)	0	0	1	0	5	1	--	--
Menarcheal (% yes)	2	0	14	6	34	35	--	--
Body Size and Composition								
Height (cm)	143.5	142.7	149.7	149.2	155.6	155.5	0.77	0.46
Weight (kg)	40.5	39.5	45.1	44.2	49.9	49.8	0.90	0.75
BMI (kg/m^2^)	19.5	19.3	20.0	19.8	20.5	20.5	0.28	0.26
Fat Mass (%)	26.1	25.4	25.6	25.3	24.7	24.7	0.62	0.57
Lean Mass (%)	71.2	71.8	71.5	71.9	72.2	72.3	-0.58	0.55

^A ^Net difference between change in intervention and control troop-level means, I = intervention troops; C = Control troops

Girls' average age was 10.5 years, 43% were categorized as Tanner stage 1 for breast development, and 1.4% were menarcheal. Calcium intake at baseline averaged 1265 mg/day. Average total energy expenditure in WBPA was 340 kcal/day and WBPA weighted MET score averaged 488, similar to the PACI scores for moderate-to-vigorous PA among 5th grade girls in a school-based nutrition and PA intervention. [24,41] At baseline, control troop mean age was significantly higher than the intervention group mean (p < .05). However, the age differences were not clinically or practically meaningful.

### Process Evaluation

Table 3 shows data on the intervention troop leader implementation (intervention fidelity) and troop participation in intervention activities (n = 15 troops). Troop leader implementation was consistently high across all troops. Troop participation levels showed some variability. However, no troops had poor participation when considering participation and exposure to all of the intervention components. The intervention component with the highest participation was troop meeting attendance (ranging from 65% to 94% in 5th grade, and 61% to 97% in 6th grade). The intervention component with the lowest participation was use of the program website (repeated use among 50% of girls in the 5th grade, and 24% of the girls in 6th grade).

**Table 3:** Process evaluation for CAL-Girls intervention troops (n = 15)

**Intervention participation**	5^th ^grade		6^th ^grade	
Website	Mean	Range	Mean	Range

Logged in at least once (%)	81.6	42.9–100.0	46.5	9.1–75.0
Logged in more than once (%)*	50.1	10.0–100.0	24.9	0.0–60.0
Posted at least one message on bulletin board (%)	46.5	7.1–100.0	7.6	0.0–62.5
Number of website hits	34.6	12.3–89.9	13.5	0.8–117.5
Number of quizzes completed	0.87	7.7–2.4	--	--
Number of Puzzled Patty activities completed	--	--	0.64	0.0–3.1
Camp attendance (%)	45.4	22.2–77.8	--	--
Proportion of troop attending meetings (%)	84.9	65.5–94.0	81.7	61.1–97.6
Proportion of troop completing home activities (%)	51.8	32.2–91.7	44.3	18.5–73.8
**Intervention fidelity**				
Troop activities completed (%)	86.1	74.2–96.9	80.2	61.2–89.3
Leaders followed guide all or most of time (%)	95.9	75.0–100.0	95.3	66.7–100.0
Leaders encouraged girls to continue behaviors outside of troop (%)	85.6	60.0–100.0	72.4	33.3–100.0

*Variable not included in website factor score.

### Effects of the Intervention on Bone Mass Gains

**Figure 1:**
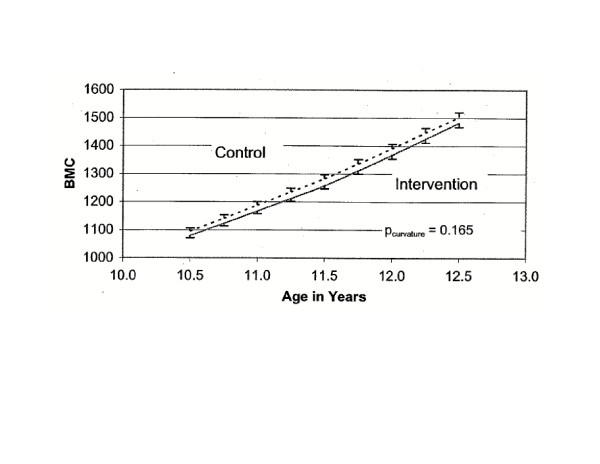
Whole Body: BMC (2_x _SE bars). CALGIRLS Study.

**Figure 2:**
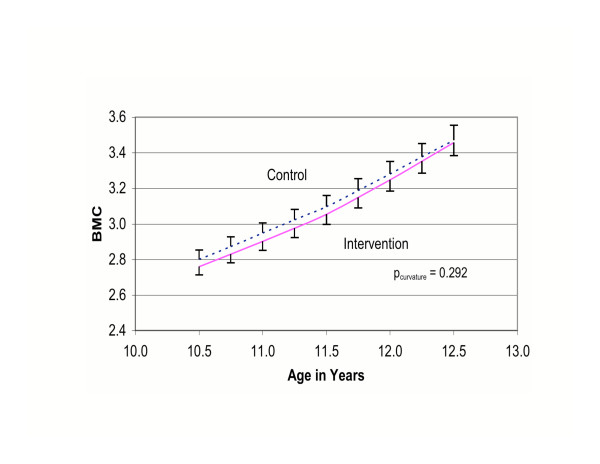
Femoral Neck of R. Hip: BMC (2_x_SE bars). CALGIRLS Study

Table 4 shows treatment-related changes in bone mass for each bone site. Treatment group differences in change in BMC were not significant for total body, total hip, femoral neck or 1/3 distal radius. Changes in total body BMC slope over time by treatment group (Figure 1) and in femoral neck BMC slope over time by treatment group (Figure 2) show similar increases among both the intervention and control groups.

**Table 4:** Baseline and follow-up dietary, physical activity, and bone variables among 30 Girl Scout troops (troop-level means) by treatment group (n = 15 control, n = 15 intervention)

**Label**	**Baseline**		**Follow-up 1**		**Follow-up 2**		**Two-year Intervention Effect**	
	C	I	C	I	C	I	Effect^A^	SE

Dietary Intake								
Calcium (mg)*	1274	1199	1245	1401	1310	1394	92	82
Energy (kcal)	2139	2136	2108	2138	2127	2047	--	--
Physical Activity								
Weight-bearing PA score@	507	448	539	472	555	531	49	91
Osteogenic score$	29	24	25	23	25	22	--	--
Bone Mineral Area (BA)								
Total body (cm^2^)	1287	1274	1443	1421	1600	1606	8.1	7.2
Total hip (cm^2^)	24.1	24.0	26.3	26.3	28.3	28.2	-0.26	0.11
Spine (L1-4) (cm^2^)	38.6	38.2	42.7	42.1	46.7	46.7	.25	.33
Femoral neck (cm^2^)	4.0	4.0	4.2	4.2	4.4	4.5	.08	.03
1/3 distal radius (cm^2^)	2.1	2.1	2.2	2.2	2.3	2.3	.01	.01
Bone Mineral Content (BMC)								
Total body (g)	1106	1084	1292	1241	1495	1485	-4.9	14.5
Total hip (g)	17.9	17.3	20.9	20.4	24.2	23.7	-.63	.29
Spine (L1-4) (g)	26.0	25.3	31.6	30.4	38.1	37.8	-.06	.58
Femoral neck (g)	2.8	2.8	3.1	3.1	3.5	3.5	.03	.04
1/3 distal radius (g)	1.1	1.1	1.2	1.2	1.3	1.3	.01	.01
Bone Mineral Density (BMD)								
Total body (g/cm^2^)	0.85	0.85	0.89	0.87	0.93	0.92	-.003	.01
Total hip (g/cm^2^)	0.74	0.72	0.79	0.77	0.85	0.83	-.010	.01
Spine (L1-4) (g/cm^2^)	0.67	0.66	0.73	0.71	0.81	0.80	-.011	.02
Femoral neck (g/cm^2^)	0.71	0.69	0.75	0.72	0.80	0.78	-.008	.007
1/3 distal radius (g/cm^2^)	0.52	0.51	0.54	0.54	0.57	0.57	.001	.004

^A ^Adjusted net difference between change in intervention and control troop-level means (adjusted for baseline age, race, weight; baseline and change in height, menarcheal status, Tanner breast stage).

* Adjusted for energy intake; and above variables, not including height or change in height)

@ Calculated variable of total weight-bearing physical activity minutes, MET value, and self-reported intensity.

Adjusted for above variables (in A), not including energy intake, height or change in height.

$ Calculated variable of ground reaction force value × (Ln (minutes in activity +1)

### Effects of the Intervention on WBPA and Dietary CA Intake

Table 4 shows adjusted changes in WBPA and dietary CA intake by treatment group. Overall, girls increased their CA intake and their WBPA over the two-year intervention. No significant treatment-related differences in change in WBPA were observed. Increases in dietary CA were significantly greater among intervention troops compared to control troops over the two-year period. However, both intervention and control troops remained at or near recommended CA levels throughout the study period.

## Discussion

The results of the present study showed that a community-based behavioral intervention to increase dietary CA intake and WBPA among 9–11 year old girls enrolled in the Girl Scouts program was not effective in increasing bone mass gains, or frequency of WBPA, over a two-year period. Significant increases in dietary CA intake were observed as a result of the intervention. However, all troops were close to recommended dietary CA levels throughout the study period. These null results were observed despite the documented high levels of troop intervention implementation and participation. Nevertheless, these results provide data that may be useful in designing future dietary and PA behavioral interventions that target youth in a community setting.

Reasons for the lack of significant effects of the intervention on behavior or bone changes are not clear. It is not surprising that no significant increases in bone mass changes were observed as a result of the intervention, since no significant changes in WBPA were produced, and CA intake was at recommended levels at baseline. Perhaps most surprising is the lack of behavior change observed among intervention troops, despite the high level of implementation and participation in the intervention activities. The troop leaders assigned to the intervention were well trained, motivated and thorough in their implementation of the intervention program activities during the troop meetings. Participation among girls was consistently high. The intervention activities comprised a significant portion of the troop meeting time for a two-year period.

These results suggest that a more structured intervention may be needed to impact WBPA changes when implemented in a community-based setting. Previous PA interventions that targeted bone mass changes among children were conducted using a more controlled intervention design. PA increases were achieved through supervised PA programs with a standardized frequency, duration and type of PA. [16,18-20,22,42,43] By contrast, the behavioral intervention program implemented in the present study focused on educational activities, and behavioral skill-building such as goal-setting, self-monitoring and incentives. A more structured intervention, one that increases the frequency, intensity and duration of WBPA, may be needed to increase WBPA enough to impact bone mass gains. This type of structured intervention may be more easily implemented in a school-based setting in which daily physical education classes are part of the normal schedule. [18,19,22,42] In addition, greater involvement of parents in structuring the home environment to increase WBPA and CA-rich food opportunities for their child may be needed to increase the magnitude of the intervention-related behavior changes and thereby increase bone mass gains. These more structured environmental intervention components may be coupled with a behavioral change intervention component for maximum effects on behavior change.

Baseline levels of CA intake and WBPA may also be important factors that influence the magnitude of the intervention effects on behavior changes. The girls in the present study had high baseline levels of CA intake, thus making further increases more challenging compared to a population with lower initial CA levels. Moreover, the high mean CA intake at baseline approached recommended intake for this age group (NIH, 1994). Additional CA may not have been associated with additional gains in bone mass even if the intervention was successful in producing further increases in CA intake. For the WBPA intervention component, a more frequent, structured, and supervised PA program and or a program that focuses on specific types of PA that are the most osteogenic, such as jumping, may prove more effective in increasing BMC gains.

Strengths of the present study include its strong study design, high level of implementation fidelity and participation, high-quality measurement of outcomes, and high levels of participant retention in the evaluation cohort. An additional strength was its implementation in a community-based youth group by trained community-based volunteers. Potential weaknesses include the use of a population with initially high levels of one of the behaviors targeted for increase (CA intake); lack of structure in the WBPA behavioral component; the use of an intervention without a strong environmental change component; and the reliance on self-report measures of PA.

The present study found that despite high levels of program implementation and participation, a community-based behavioral intervention to increase bone mass gains in 9–11 year old girls was not successful in changing bone mass outcomes or WBPA. Future research is needed to further explore the effectiveness of community-based behavioral interventions for dietary CA and WBPA increases that target children. However, a useful prelude to such research would be a series of additional controlled studies that examine physical activity dosages and the types of activities that produce the strongest effects on bone mass gains in targeted age groups. [20] A similar set of systematic dosage studies for change in CA intake in populations that have an initially low or high CA level are needed. Together, the results of such studies could inform a more focused and potentially more effective behavioral intervention for bone mass gains targeting children that could be implemented in a free-living setting in the community.

## Competing interests

The author(s) declare that they have no competing interests.

## Authors' contributions

SAF developed and directed the overall study and wrote the manuscript. MS contributed to the study design, direction and implementation and manuscript writing. JAF was the project director responsible for daily oversight of intervention evaluation activities and contributed to the manuscript writing. JHH contributed to the project design and oversight, data analysis and manuscript writing. PH contributed to the study design, data analysis and manuscript writing. DNS contributed to the study design, development and manuscript writing. KE contributed to the manuscript writing.
